# Olaparib for metastatic breast cancer in a patient with a germline *PALB2* variant

**DOI:** 10.1038/s41523-020-00174-9

**Published:** 2020-07-24

**Authors:** Sherko Kuemmel, Hakima Harrach, Rita K. Schmutzler, Athina Kostara, Katja Ziegler-Löhr, Mark H. Dyson, Ouafaa Chiari, Mattea Reinisch

**Affiliations:** 1 Interdisciplinary Breast Unit, Kliniken Essen-Mitte, Essen, Germany; 2 Center for Familial Breast and Ovarian Cancer, Center for Integrated Oncology (CIO), University of Cologne, Faculty of Medicine and University Hospital Cologne, Cologne, Germany; 3 Schwerpunktpraxis für Gynäkologische Onkologie, Cologne, Germany

**Keywords:** Cancer therapy, Breast cancer

## Abstract

There is a strong biologic rationale that poly(adenosine diphosphate-ribose) polymerase (PARP) inhibitors may benefit a broader range of metastatic breast cancer (MBC) patients than covered by current approvals, which require a germline *BRCA1/2* sequence variant affecting function. We report a patient with germline/somatic *BRCA1/2* wild-type MBC, who had a dramatic response to the PARP inhibitor olaparib of at least 8 months’ duration. The patient is a 37-year-old woman with recurrent, hormone receptor-positive, HER2-negative MBC that had progressed despite hormonal therapy and palbociclib. Sensitivity to olaparib was likely conferred by a germline sequence variant affecting function in *PALB2* (exon 1, c.18G>T, p.(=)). This case documenting activity of olaparib monotherapy in germline/somatic *BRCA1/2* wild-type MBC illustrates that the clinical potential of PARP inhibition in MBC extends beyond currently approved indications to additional patients whose tumors have (epi)genetic changes affecting homologous recombination repair.

## Introduction

The poly(adenosine diphosphate-ribose) polymerase (PARP) inhibitors olaparib and talazoparib are approved for treatment of hormone receptor (HR)-positive or triple-negative advanced breast cancer in patients with germline variants affecting function of breast cancer susceptibility genes 1 or 2 (*BRCA1/2*). The approved indications derive from the eligibility criteria for the randomized Phase 3 trials of these agents versus a variety of standard chemotherapy options in this setting, which required central verification of germline *BRCA1/2* sequence variation^1,2^. Selection of this target population was based on the homologous recombination deficiency (HRD) caused by loss of functional BRCA1/2, which renders cancer cells unable to accurately repair the double-strand DNA breaks induced by PARP inhibitors^3^. Nonetheless, according to this biologic rationale, the spectrum of breast cancers with the potential to respond to PARP inhibitors may extend beyond patients with germline *BRCA1/2* sequence variants to include those with germline or somatic sequence variants, or epigenetic inactivation, of these or other genes involved in maintenance of genomic integrity^3^.

We present the case of a patient with metastatic breast cancer (MBC) without a germline or somatic *BRCA1/2* sequence variant, but with a germline sequence variant affecting function in partner and localizer of BRCA2 (*PALB2*), who exhibited a dramatic response to olaparib. This case illustrates the clinical potential of PARP inhibitors in MBC outside currently approved indications.

## Results

### Case history and presentation

In October 2013, a 31-year-old woman was diagnosed with estrogen receptor-positive (70%), progesterone receptor-positive (15%), human epidermal growth factor receptor-2 (HER2)-negative, high grade (G3) invasive cancer of the right breast (pT1c, pN1a, M0; Ki-67 index 20%), with concurrent ductal carcinoma in situ (DCIS). Primary treatment was subcutaneous right mastectomy with implant-based reconstruction, adjuvant chemotherapy (epirubicin/cyclophosphamide and docetaxel), irradiation of the affected breast and supra- and infraclavicular nodes, and adjuvant tamoxifen, which was discontinued after 8 weeks due to a major depressive episode. Thereafter, the patient remained relapse free for 2 years, until positron emission tomography (PET)/computed tomography (CT) revealed local recurrence in the thoracic wall with mediastinal nodal involvement with no other distant metastases. Salvage mediastinal lymphadenectomy was performed, but the patient initially declined further systemic therapy. However, 14 months later, local recurrence, distant bone and liver metastasis, and deteriorating clinical condition led to initiation of palliative therapy with letrozole, a gonadotropin-releasing hormone analog, palbociclib, and denosumab. Partial radiologic response (per Response Evaluation Criteria in Solid Tumors (RECIST) v1.1) was attained under therapy until PET/CT after 11 months indicated progression of bone metastases and a new pericardial lesion.

### Genetic analysis

The patient reported no family history of breast cancer at initial diagnosis and tested negative for germline sequence variants in *BRCA1*, *BRCA2, CHEK2*, and *RAD51C*. However, subsequent genetic analysis by next-generation sequencing (NGS) identified a germline sequence variant in *PALB2* (exon 1, c.18G>T, p.(=)) of class IV (i.e., likely pathogenic). Although the patient’s mother was diagnosed with DCIS during the course of disease, genetic profiling of the mother indicated that the patient’s germline variant had in fact been paternally inherited. NGS analysis of mediastinal nodal tumor tissue acquired at relapse indicated a microsatellite-stable tumor with low mutational burden (4 mut/Mb), *ARID1A* mutation (Q1409*), and amplification of *RAD21* and *RPOR*, but yielded no clinically actionable findings. We had limited therapeutic options in this setting due to patient refusal of palliative chemotherapy. Given the well-established efficacy of PARP inhibitors in patients with germline *BRCA1/2* sequence variants^1,2^ and the phenotypic similarities between *PALB2*-mutant and *BRCA1/2*-mutant cells^4^, we reasoned that olaparib could provide clinical benefit for this patient with a germline *PALB2* variant and HR-positive, HER2-negative MBC that had progressed despite endocrine and cyclin-dependent kinase-4/6 inhibitor therapy.

### Management

**Fig. 1:**
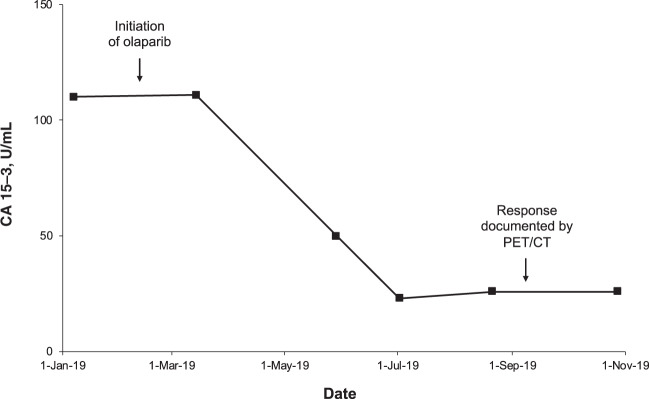
Tumor marker response to olaparib. Compared with pretreatment levels, there was a marked reduction in circulating CA 15–3 at 3 months after initiation of olaparib, which was deepened and sustained after 8 months of treatment.

**Fig. 2:**
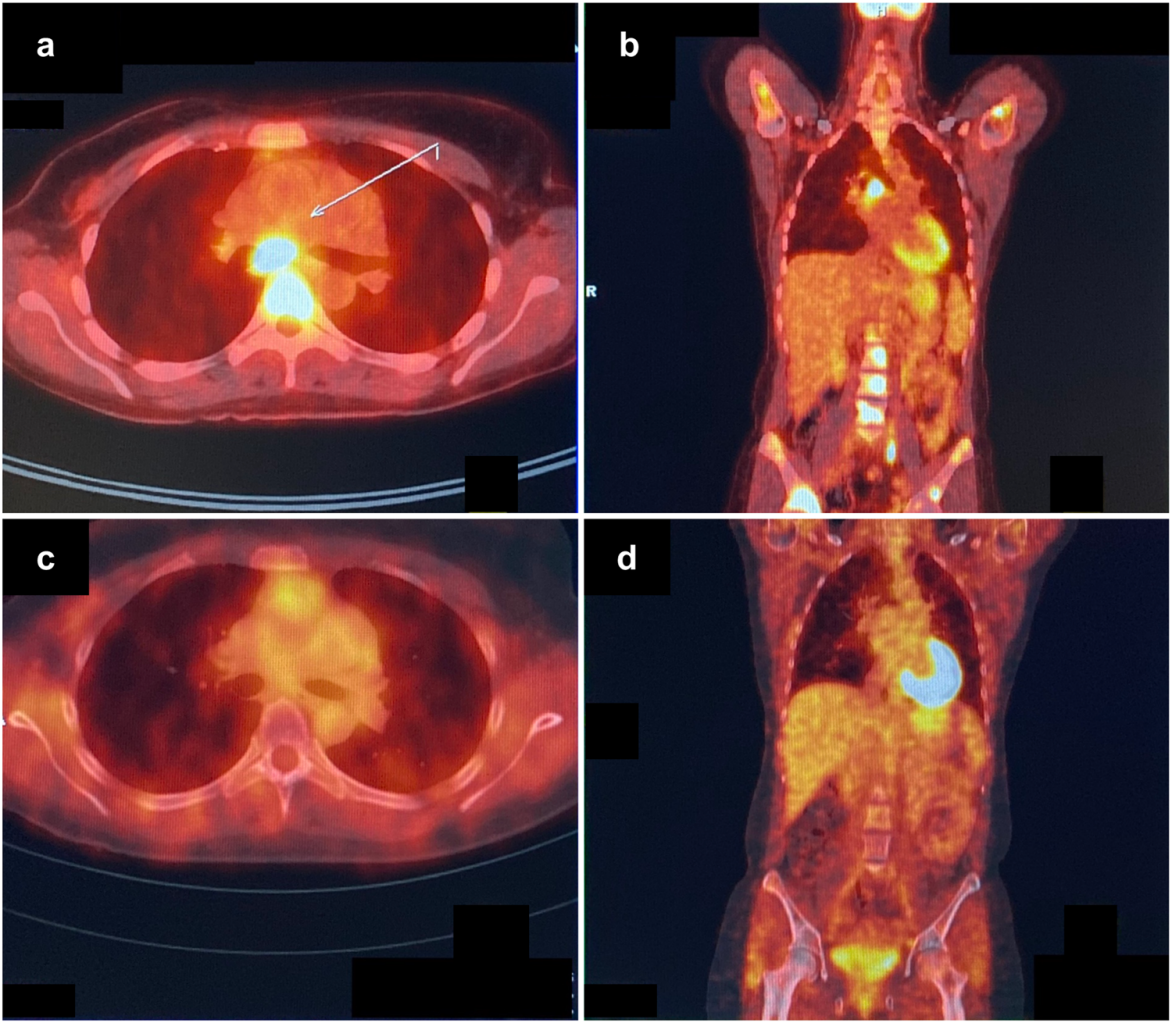
Radiologic response to olaparib. Axial (**a**) and coronal (**b**) PET/CT images acquired prior to initiation of olaparib show hypermetabolic mediastinal lymph nodes and multifocal bony attachment in the spine and pelvis. After 7 months of olaparib therapy, axial (**c**) and coronal (**d**) PET/CT images indicate significant remission of lymph node and bone metastases.

In February 2019, off-label treatment with olaparib was initiated at a dose of 600 mg/day (two 150-mg tablets twice daily), alongside continuing therapy with denosumab. The olaparib dose was reduced to 200 mg/day (one 100-mg tablet twice daily) after 3 weeks to manage side effects of nausea, vomiting, and fatigue that had not responded to supportive therapy. Within a few weeks, the patient reported an improvement in well-being, with a reduction in pain, increased appetite, and enhanced quality of life. There was a substantial reduction in tumor marker CA 15–3 by month 3 that was sustained after 8 months of therapy (Fig. 1). Based on patient preference, the first restaging was conducted 7 months after initiation of olaparib by PET/CT (Fig. 2). The scan demonstrated significant regression of all lesions, corresponding to a partial response per RECIST v1.1. The only remaining PET-positive lesions consisted of a left inguinal lymph node and low residual activity of skeletal metastases in the thoracic spine and the right sacrum and ilium. After 8 months of therapy, the patient remains in good clinical condition, continues to receive olaparib, and will be followed-up with regular tumor marker assessment and PET/CT.

## Discussion

This case report documents response to olaparib monotherapy in a patient with MBC with a sequence variant affecting function in *PALB2*. Like *BRCA1/2, PALB2* encodes a homologous recombination repair protein whose loss of function produces synthetic lethality in combination with PARP inhibition^4^. Sensitivity to olaparib may, therefore, be attributable to this *PALB2* variant^5,6^. In addition, the tumor showed a loss-of-function alteration in the chromatin remodeling complex subunit *ARID1A*, which also leads to defective double-strand break repair and vulnerability to PARP inhibition^7^. While variants in *PALB2* and *ARID1A* are implicated in only a small proportion of breast cancers (0.5–1% and 4%, respectively)^8–10^, this case provides proof-of-concept of the therapeutic potential of PARP inhibitors in a wider range of MBC patients than covered by current approvals. In a large series of unselected breast cancers, 16% of tumors had inherited or acquired driver sequence variants across a panel of homologous recombination repair genes, including *BRCA1/2*, *PALB2*, *ARID1A*, *ATM*, *CHEK2*, and *BAP1*^10^. A higher proportion of breast cancers (>20% overall; up to 70% in triple-negative disease) test positive in assays that detect genomic scars characteristic of HRD, irrespective of the underlying (epi)genetic mechanism^11,12^.

Our patient showed a duration of response of at least 8 months (ongoing at the time of writing), which compares favorably with the median progression-free survival of 7.0 months with olaparib in the Phase 3 OlympiaD trial^1,13^. That study also provided evidence of overall survival benefit versus chemotherapy among patients who, like the present case, had not received prior chemotherapy for metastatic disease^13^. While the published Phase 2/3 olaparib and talazoparib MBC trials have focused on patients with germline *BRCA1/2* sequence variants^1,2,14,15^, several observations suggest a broader range of patients with MBC could benefit. For example, complete response was previously reported in a patient with wild-type germline *BRCA1/2* status in a Phase 1 olaparib study^16^. However, the response cannot be unambiguously attributed to olaparib due to the coadministration of paclitaxel and, as far as we are aware, no further germline or somatic genetic profiling of this patient has been reported. Clinical activity of olaparib monotherapy has also been documented in a patient with triple-negative MBC with brain metastasis and a somatic sequence variant in *BRCA1*^17^. Results reported in abstract form describe response to talazoparib in two patients with wild-type germline/somatic *BRCA1/2* MBC and a germline *PALB2* variant^18^. Recently, Grellety et al. also described a marked response to olaparib in a patient with HR-positive, HER2-negative, *BRCA1/2* wild-type MBC, and a germline variant in *PALB2* different from the one detected in the present case, as well as a second *PALB2* mutation in the tumor^19^. In addition, subgroup analyses of Phase 2 trials provide evidence for the efficacy of PARP inhibitors in combination with neoadjuvant chemotherapy in early breast cancer with wild-type germline *BRCA1/2* (I-SPY 2)^20^ or wild-type germline and somatic *BRCA1/2* and HRD (GeparOLA)^21^. Beyond breast cancer, trials in advanced ovarian cancer and metastatic castration-resistant prostate cancer have demonstrated efficacy of various PARP inhibitors in populations other than carriers of germline sequence variants in *BRCA1/2*, including somatic *BRCA1/2* variant-associated cancers, and tumors with or without HRD^22,23^.

Female heterozygous carriers of sequence variants affecting function in *PALB2* have a cumulative breast cancer risk of 14% by age 50 years and 35% by age 70 years^5^. The current case is consistent with the typical presentation of breast cancers in *PALB2* sequence variant carriers, which frequently show high nuclear grade and mitotic index, with estrogen receptor-positivity (53–74%; although triple-negative disease is significantly overrepresented among carriers of pathogenic *PALB2* variants)^5,24,25^. The observed *PALB2* variant (exon 1, c.18G>T, p.(=)) is a synonymous change that leads to abnormal transcript splicing and loss of function via premature termination of translation^26^. Given the poor prognostic impact of germline *PALB2* sequence variants and limited evidence regarding systemic therapy in this subgroup^25^, the marked response in the present case is particularly noteworthy.

In conclusion, this case report documenting response to olaparib of at least 8 eight months’ duration in a patient with progressive MBC lacking germline or somatic *BRCA1/2* sequence variants illustrates the clinical potential of olaparib monotherapy in MBC beyond currently approved indications. Taken together with the biologic rationale for synthetic lethality of PARP inhibition in tumors with HRD^3^, these data suggest that additional patients with relapsed and/or metastatic disease could benefit from this therapeutic strategy. We keenly await the results of ongoing clinical trials of PARP inhibitors in broader MBC populations (e.g., NCT03344965, NCT02401347, and NCT02029001), including patients with germline or somatic sequence variants in other HRD driver genes or HRD-high status in genomic assays.

## Methods

### Ethics statement

Off-label treatment with olaparib in this patient with MBC lacking germline *BRCA1/2* sequence variants affecting function was provided as compassionate use in the palliative setting in line with national regulations and did not require ethics committee approval. The patient provided written informed consent prior to initiation of treatment. Written informed patient consent was also obtained for publication of the data contained in this case report.

### Genetic analyses

Germline genetic analysis was conducted by NGS using the TruRisk^®^ Version 2 gene panel, which includes 15 core genes: *ATM*, *BRCA1*, *BRCA2*, *BRIP1*, *CDH1*, *CHEK2*, *MLH1*, *MSH2*, *MSH6*, *PALB2*, *PMS2*, *PTEN*, *RAD51C*, *RAD51D*, and *TP53*. Genomic DNA was extracted from blood samples, enriched for target sequences with the SureSelect^XT^ kit (Agilent Technologies, Inc., Santa Clara, CA, USA), and directly sequenced using the NextSeq platform (Illumina, Inc., San Diego, CA, USA). For tumor genetic analysis, DNA was extracted from formalin-fixed, paraffin-embedded tumor samples and analyzed using a commercial NGS assay (FoundationOne^®^ CDx, Foundation Medicine, Inc., Cambridge, MA, USA).

### Reporting summary

Further information on experimental design is available in the Nature Research Reporting Summary linked to this article.

## Supplementary information

Supplementary Information

## Data Availability

The data generated and analyzed in this study are described in the following metadata record: 10.6084/m9.figshare.12370346^27^. The tumor DNA sequencing data have been deposited in NCBI Sequence Read Archive and are available via accession: https://identifiers.org/ncbi/bioproject:PRJNA635377^28^. The tumor DNA NGS analysis is contained in the spreadsheet Tumor DNA analysis.xlsx, which is included with this data record^27^; this file summarizes the mutant allele frequency and copy number variations that were detected by NGS. The germline DNA sequencing cannot be shared due to German data protection law considerations designed to safeguard patient anonymity. However, the authors confirm there were no other rare germline variants detected besides the reported *PALB2* variant, which has previously been described and is available in dbSNP under this accession: https://identifiers.org/dbsnp:rs587782462^29^. The data underlying Fig. 1 in the related manuscript are contained in the spreadsheet CA 15-3.xlsx, included with this data record^27^.
